# Habitual- and Meal-Specific Carbohydrate Quality Index and Their Relation to Metabolic Syndrome in a Sample of Iranian Adults

**DOI:** 10.3389/fnut.2022.763345

**Published:** 2022-04-01

**Authors:** Maryam Majdi, Hossein Imani, Elham Bazshahi, Fatemeh Hosseini, Kurosh Djafarian, Azadeh Lesani, Zahra Akbarzade, Sakineh Shab-Bidar

**Affiliations:** ^1^Department of Community Nutrition, School of Nutritional Sciences and Dietetics, Tehran University of Medical Sciences, Tehran, Iran; ^2^Department of Clinical Nutrition, School of Nutritional Sciences and Dietetics, Tehran University of Medical Sciences, Tehran, Iran

**Keywords:** carbohydrate quality index, metabolic syndrome, meal-specific, habitual meal, cross-sectional study

## Abstract

**Aim:**

Most studies on diet quality have focused on the habitual and overall intake of foods without considering intakes at specific eating occasions. This study aimed to assess the association between habitual- and meal-specific carbohydrate quality index (CQI) and metabolic syndrome (MetS) in Iranian adults.

**Methods:**

In this cross-sectional study, data from 850 participants were analyzed. Dietary information was obtained from a 3-day nonconsecutive 24 h recall. CQI was calculated from three criteria: dietary fiber, glycemic index, and solid carbohydrate/total carbohydrate ratio. The association between CQI and MetS was assessed by logistic regression.

**Results:**

The prevalences of MetS in the lowest and highest tertile of CQI were 30.1 and 33.7, respectively (*P* = 0.6). In habitual diet and all the three meals, we failed to find any significant association between tertiles of CQI and MetS either before or after adjustment for covariates. However, in the habitual meals [odds ratio (OR): 0.69, 95% CI: 0.47–0.96] and lunch meals (OR: 0.66; 95% CI: 0.47–0.94), the highest CQI in comparison to the lowest one, significantly decreased the low high-density lipoprotein (HDL). In addition, the trend of low-HDL with CQI in habitual meal and lunch meal was statistically significant.

**Conclusion:**

The results of this study showed that CQI was not associated with MetS and its components. Further investigations into the mechanisms underlying the role of carbohydrate quality in developing metabolic disorders are warranted.

## Introduction

Metabolic syndrome (MetS) deputes an interrelated metabolic disorder determined by central obesity, deviant glucose hemostasis, and lipid disorders, namely, elevated serum triglyceride (TG), low high-density lipoprotein cholesterol, and elevated blood pressure (1). The prevalence of the MetS among adults in the United States was estimated between 34.3 and 38.5% (2), and in some European countries, at least 25% (3). Recently published data from Iran show that the prevalence of MetS is 21.1% (4). Based on the evidence, a combination of genetic and environmental factors has been shown to play a role in developing this syndrome (5, 6). Among environmental factors, meal-timing, meal frequency, and dietary quality may play an essential role in cardiometabolic risk factor management (7). Meal timing can affect circadian rhythm and cardiometabolic risk factors (8–10). Epidemiological studies indicated that eating meals at the inappropriate time of the day increases the risk of obesity, type 2 diabetes, and cardiovascular disease (CVD) (11, 12). Limited studies have been performed on meal frequency (daily eating occasions) and cardiometabolic risk factors with different results. One study showed that meal frequency was inversely related to high TGs, high blood pressure, and obesity (13). Besides, individuals who take three meals/day than individuals who received one meal/day, fewer systolic and diastolic blood pressure, lower cholesterol level, and upper TG concentration have been reported (14).

In recent decades, the effort to preclude MetS has focused on declining risk factors and recommending healthy behaviors, especially healthy eating habits (15). In this context, studies and evidence have shown that improving the quality of dietary carbohydrates, instead of modulating their quantity, may have a more significant impact on modulating cardiac-metabolic risk factors (16). Most of the available evidence has examined the various components of the quality of carbohydrates received, such as fiber intake, glycemic index (GI), and glycemic load, separately (17, 18). Former studies have explored the association between consumption of carbohydrates and MetS and have indicated positive (19–21), contradictory (22), and null (23) effects. But since a single component cannot be an appropriate criteria for evaluating the quality of carbohydrate revived, border criteria that can accommodate several single components are used as the carbohydrate quality index (CQI), which is a convenient indicator of the quality of the carbohydrate intake. The CQI was defined by summing up the following criteria: GI, dietary fiber intake, whole grains to total grains ratio, and solid carbohydrates to total carbohydrates (24). Since whole grains in the Iranian diet are limited, its calculation has been abandoned in this study. Few studies have investigated the association between CQI and pathological conditions. A cross-sectional study in Ghana indicated a reverse association between CQI and abdominal obesity (25). In addition, the reverse association between COI and general obesity/overweight has been demonstrated in one prospective study (24). Researchers assayed the relation between dietary patterns based on macronutrients and blood factors such as lipid profile and fasting blood glucose. Still, there has not been much attention paid to meals (26–28). In addition, a dietary recommendation based on meals can be an effective intervention in changing inappropriate habitual intake (29).

Since carbohydrates as a major nutrient provide an essential part of the energy required of the adult population, it is assumed that carbohydrate intake may play a more prominent role in public health. Hence this study aims to assess the association between CQI of meal-specific dietary patterns and MetS and its components among Tehranian adults.

## Materials and Methods

### Data and Study Participants

This cross-sectional study was done within 25 health houses in the Tehran Metropolis. A total of 850 adult participants aged between 18 and 65 years were included. This study was conducted according to the guidelines laid down in the Declaration of Helsinki. All the procedures involving human subjects were approved by the ethical standards of the Tehran University of Medical Sciences (ethic number: IR.TUMS.MEDICINE.REC.1399.797), which approved the protocol and informed consent form. All the participants signed a written informed consent before the start of this study.

Adults with a previous history of any major illness such as myocardial infarction, diabetes, cancer, renal disease, and CVD and who were not desired to contribute to the study were excluded. In addition, those who were experiencing any special diet or diet therapy were also excluded from the study. But, in continuation of this study and review of the questionnaires were a limited number of participants who were not considered acute patients and only had a mild and controlled disease that were not excluded from this study. In this case, to prevent errors in the results of the study, individuals were adjusted in terms of having the underlying disease (Supplementary Figure 1). This study included both the genders and living in the study region and willing to participate in this study.

### Dietary Assessment, Meal Timing, and CQI Calculation

Dietary data were gathered by using repeated but nonconsecutive (Monday to Sunday) 24-h dietary recall method. The 24-h meal was structured and included breakfast, lunch, and dinner. The first 24-h recall is obtained through interviews, the other two recalls are recorded by telephone during two repeated nonrandom days during the study and information was recorded. The habitual diet was calculated using the mean of three 24-h recalls. Food and the food groups were extracted through these questionnaires. Meals usually include breakfast, lunch, and dinner. More energy content was used to classify meals. Thus the largest meal was considered between 5:00 and 11:00 as breakfast, 11:00 and 16:00 as lunch, and between 16:00 and 23:00 as dinner and the smaller intake between main meals was identified as snacks (30, 31). A habitual diet is the combination of meals and snacks which includes 4 meals and snacks throughout the day, including breakfast, lunch, evening snack, and dinner.

Finally, the total value of nutrients in all the meals and snacks was calculated daily. CQI was calculated based on the energy-adjusted amount of total carbohydrate intake values calculated using the residual method (32). CQI was defined by summing up the following four criteria: (1) ratio of solid carbohydrates to total carbohydrates, (2) dietary fiber intake (g/day), (3) GI, and (4) ratio of whole grains to total grains (whole grains, refined grains, and their products). Subjects were categorized into quintiles and take a value (ranging from 1 to 5) for each quintile according to each of these four criteria; however, the scoring of GI was reversed; thus, those in the fifth quintile received one point, and those in the first quintile received five points. Finally, an overall CQI was computed by adding all values of the four criteria (ranging from 4 to 20). It was also ranked into quintiles (24). But given that the Iranians are using whole grains in the diet is limited, this component is not calculated, so the final score ranges from 3 to 15.

Glycemic index (GI) values were obtained from international tables (33), the GI of Iranian foods (34), and literature reviews. As if food item was not available in any of the mentioned tables, we used the GI values of chemically and physically similar food items for those foods (34). Glucose was used as the reference (GI for glucose = 100). The mean of the GI values was assigned if more than one eligible GI value was available for a specific food item. The carbohydrate content of each food was determined using standard portion sizes from the United States Department of Agriculture food composition databases (35). All the nuts and vegetables except starchy roots were considered as very low GI (ranging from 10 to 20). Solid carbohydrates were obtained by subtracting the amount of liquid carbohydrate (summing up sweetened beverages and fruit juice) from total carbohydrate intake.

### Anthropometric Assessment and Biochemical Tests

Weight was measured using a Seca weighing scale (Seca and Corporation KG; 22 089 Hamburg, Germany; Model: 874 1321009; designed in Germany; made in China) with light clothing (without a coat and raincoat). A wall stadiometer board was used for participants' height without shoes with a sensitivity of 0.1 cm height measurements. Body mass index (BMI) was calculated as weight (in kilograms) divided by height (in meters squared). Waist circumference was measured according to the guiding protocol of the WHO, at the midpoint between the lower border of the rib cage and the iliac crest, using a nonstretchable fiberglass measuring tape. Eventually, the waist-hip ratio (WHR) was calculated for each person. WHR ≥0.5 was adopted for overweight and abdominal obesity for uniformity regarding age differences (36). Blood pressure is measured by a digital barometer (BC 08, Beurer, Germany) after at least 10–15 min of rest and sitting. Blood pressure was measured two times for each person, and the average blood pressure was reported for each person. Of all participants, 10 ml of fasting blood was taken between 7 and 10 a.m. in the acid-washed test tubes without anticoagulant until after room temperature maintenance for 30 min. Minute blood clots and centrifuge at 1,500 g for 20 min. The serums are poured into microclean tubes and stored in the −80°C freezer until the future test. Fasting blood sugar was assayed by the enzymatic (glucose oxidase) colorimetric method using a commercial kit (Pars Azmun, Tehran, Iran) at the sampling day. Serum total cholesterol and high-density lipoprotein cholesterol (HDL-C) were measured using a cholesterol oxidase phenol amino antipyrine method and TG was measured using a glycerol-3 phosphate oxidase phenol amino antipyrine enzymatic method on the same day after collecting all the samples.

### Sociodemographic and Lifestyle Variables

General information such as age, marital status, smoking status, living situation (alone or with someone), and disease was recorded by asking participants with a general information questionnaire registered. International Physical Activity Questionnaire is used to examine people's physical activity (37), which records three intensity levels of activity based on the metabolic equivalents (METs). METs were classified as low (<600 MET-min/week), moderate (600–3,000 MET-min/week), and vigorous (>3,000 MET-min/week).

### Metabolic Syndrome

Metabolic syndrome (MetS) and its components were defined using the following criterion (1). Individuals who have at least three or more of the following disorders were classified as having MetS: high waist circumference (≥88 for women and ≥102 for men); elevated TG levels (≥150 mg/dl); low HDL-C levels ( ≤ 50 mg/dl for women and ≤ 40 mg/dl for men); high blood pressures (systolic blood pressure ≥ 130 mm Hg and diastolic blood pressure ≥ 85 mm Hg) or use of antihypertensive medication; and high fasting glucose levels (≥100 mg/dl) or use of hypoglycemic medication.

### Statistical Analysis

Energy-adjusted dietary CQI was used to classify participants into tertiles. According to the type of variables, the comparison of quantitative mean variables between the tertiles of subject characteristics and anthropometric measurement was performed using one-way ANOVA and comparison of qualitative variables distribution between the tertiles with the chi-squared test. Logistic regression was performed to investigate the relationship between CQI as an independent variable and MetS and its components as a dependent variable in an unadjusted and multivariable-adjusted model. In this regard, age, sex, energy intake, physical activity, marital status, smoking status, educated status, underlying disease, and BMI were included as covariates in the modified regression model. All the statistical analyses were done using IBM Statistical Package for Social Sciences (V.22; SPSS Inc.), and *P* < 0.05 was considered as statistically significant.

## Results

The mean age of study participants with MetS was 46.1 ± 10 and the mean BMI with MetS was 29.2 ± 4.71. The prevalence of MetS among participants in the lowest and highest tertiles of CQI were 30.1 and 33.7, respectively (*P* = 0.6). The mean CQI in participants with MetS was 9.15 ± 2.83 (Supplementary Table 1).

Among the participants, 30, 24, and 5 participants were removed from breakfast, lunch, and dinner, respectively, due to the lack of enough information for the final analysis. As a result, 820, 826, and 845 participants remained in the study for final analysis at breakfast, lunch, and dinner meals.

General characteristics of study participants according to carbohydrate quality score based on habitual diet and meal is given in Table 1. Within lunch meals, those in the top tertiles of CQI were less likely to be current smokers (*P* = 0.05). In habitual diet and all the three meals, the distribution of participants in terms of other general characteristics across tertiles of CQI was not significantly different.

**Table 1:** General characteristic of study participants according to tertiles (T) of carbohydrate quality index (CQI).

	**CQI (breakfast)**				**CQI (lunch)**				**CQI (dinner)**				**CQI (habitual)**			
	**T1** **(3–7)**	**T2** **(7–11)**	**T3** **(11–15)**	P^**^	**T1** **(3–7)**	**T2** **(7–11)**	**T3** **(11–15)**	P^**^	**T1** **(3–7)**	**T2** **(7–11)**	**T3** **(11–15)**	P^**^	**T1** **(3–7)**	**T2** **(7–11)**	**T3** **(11–15)**	P^**^
Participant	273	274	273		275	276	275		281	282	282		276	277	276	
**Sex**																
Male%	30	40	30	0.1	37.7	33.3	29	0.3	32.9	32.3	34.8	0.9	27.8	31.9	40.3	0.1
Female%	33.9	31.9	34.2	0.1	32.3	33.7	34	0.3	33.3	33.6	33	0.9	34.5	33.7	31.8	0.1
Age	42.7 ± 11	42.2 ± 10	42 ± 11.6	0.7	42.3 ± 11	42.7 ± 10.8	42 ± 11	0.7	41.5 ± 10.7	43.3 ± 10.9	42.1 ± 11	0.1	41.8 ± 10.7	42.5 ± 10.6	42.4 ± 10.4	0.7
Educated	33.3	33.8	32.9	0.6	33.7	32.5	33.8	0.03	33.4	33.2	33.4	0.9	33.6	33.6	32.8	0.7
Marital status (married %)	32.6	34.9	32.5	0.2	33.2	34.1	32.7	0.8	34.2	33.1	32.7	0.4	32.9	33.8	33.2	0.7
Smoking (smoker %)	23.3	37.2	39.5	0.3	50	30.6	19.4	0.05	32.6	34.9	32.6	0.9	34.1	36.6	29.3	0.8
Underlying disease (yes %)	35.8	31.3	32.8	0.3	31	34.8	34.2	0.4	31.7	34.8	33.4	0.6	32.5	34.3	33.1	0.8
**Activity score**																
Low	34.1	32.5	33.4	0.9	34.2	32.4	31.6	0.3	32.1	34.3	33.6	0.5	34.6	33.9	31.6	0.3
Moderate	32.5	34.7	32.8	0.9	32.4	32.4	35.3	0.3	34.8	32.6	32.6	0.5	33.9	32.9	33.2	0.3
High	28.8	34.2	37	0.9	31.6	30.3	38.2	0.3	35.1	32.5	32.5	0.5	24.3	32.4	43.2	0.3
BMI	27.6 ± 7.31	27.2 ± 4.63	27.1 ± 4.63	0.5	27.4 ± 6.84	27 ± 5.27	27.4 ± 5.27	0.6	27.1 ± 4.18	27.7 ± 7.04	27.1 ± 5.3	0.2	27 ± 4.42	27.3 ± 4.52	27.3 ± 4.59	0.6
Weight (kg)	72.4 ± 14.6	72.3 ± 13.6	71.8 ± 13.5	0.8	72.5 ± 13.8	71.6 ± 13.9	72.1 ± 13.9	0.7	71.8 ± 11.6	72.5 ± 14.7	72 ± 15.1	0.8	71.6 ± 13	72.71 ± 4.5	72.8 ± 13.5	0.5
Height (cm)	162.4 ± 9.88	163 ± 8.84	162.5 ± 8.38	0.7	162.9 ± 9.51	162.6 ± 8.13	162.3 ± 9.46	0.7	162.8 ± 8.76	162.1 ± 9.34	162.9 ± 8.89	0.4	162.2 ± 9.13	162.8 ± 8.5	162.9 ± 8.9	0.5
WC (cm)	89.4 ± 12.3	89 ± 12.9	89.1 ± 12.1	0.9	89.5 ± 12.1	88.6 ± 11.4	89.1 ± 12.7	0.6	88.8 ± 11.1	89.2 ± 12.1	89.3 ± 13	0.8	88 ± 11.6	90 ± 12	89.6 ± 11.5	0.09

*Values are means ± standard deviations (SD) or percentages*.

*Chi-square test used for categorical variables, one-way ANOVA for continuous variables*.

** *P < 0.05*.

*^‡^Underlying disease: Including diabetes, hypertension, dyslipidemia, cardiovascular disease, stroke, cancer, respiratory disease, and osteoporosis*.

*BMI, body mass index; WC, waist circumference; Kg, kilogram; cm, centimeter*.

The evaluation of biochemical biomarkers showed no significant statistical differences in laboratory characteristics across tertiles of CQI in habitual meals and all the three meals (Table 2).

**Table 2:** Laboratory results of study participants according to tertiles (T) of CQI.

	**CQI (breakfast)**				**CQI (lunch)**				**CQI (dinner)**				**CQI (habitual)**			
	**T1** **(3–7)**	**T2** **(7–11)**	**T3** **(11–15)**	P^**^	**T1** **(3–7)**	**T2** **(7–11)**	**T3** **(11–15)**	P^**^	**T1** **(3–7)**	**T2** **(7–11)**	**T3** **(11–15)**	P^**^	**T1** **(3–7)**	**T2** **(7–11)**	**T3** **(11–15)**	P^**^
Participant	273	274	273		275	276	275		281	282	282		276	277	276	
FBG (mg/dl)	108.1 ± 9.35	107.3 ± 35.5	107.7 ± 35.9	0.96	108.5 ± 35.2	108.6 ± 42.5	106.2 ± 28.4	0.67	103.8 ± 24.7	109.2 ± 44.3	110.5 ± 5.35	0.06	108 ± 38.5	107.6 ± 40.5	107.2 ± 25.1	0.96
TG (mg/dl)	152.8 ± 85	139.5 ± 73.6	141.9 ± 73.7	0.09	147.3 ± 81.8	146.8 ± 75.8	140.7 ± 75.4	0.54	141.8 ± 69.7	147.1 ± 82.5	145.4 ± 1.80	0.7	142.6 ± 75.9	142.7 ± 78.3	149.4 ± 78.9	0.49
TC (mg/dl)	193.7 ± 41.2	199.1 ± 48.3	195.8 ± 45.1	0.35	197.7 ± 44.7	195.5 ± 43.3	194.8 ± 46.9	0.73	196.4 ± 44.7	196 ± 47.4	195.4 ± 5.42	0.9	193.8 ± 43.1	194 ± 45.9	201.1 ± 44.9	0.09
HDL-C (mg/dl)	49.5 ± 10.6	50.5 ± 9.88	49.6 ± 10.1	0.46	49.5 ± 10.4	49.5 ± 10	50.5 ± 10	0.4	49.9 ± 10.1	50.1 ± 10.2	49.5 ± 10.2	0.8	49.4 ± 9.95	49.5 ± 9.99	50.7 ± 10.6	0.23
SBP (mmHg)	118.6 ± 15.1	116.4 ± 14.2	118.8 ± 16.3	0.11	116.5 ± 14.7	119 ± 15.4	118.4 ± 15.4	0.12	117.7 ± 15.4	119.1 ± 14.9	117.3 ± 15.4	0.34	117.9 ± 15.9	116.9 ± 14.4	119.2 ± 15.4	0.19
DBP (mmHg)	79.2 ± 9.23	77.8 ± 9.08	79.2 ± 10.7	0.15	78.4 ± 9.46	78.8 ± 10	78.9 ± 9.6	0.78	78.9 ± 9.35	79.1 ± 10.4	78 ± 9.38	0.35	78.7 ± 9.69	78.5 ± 8.89	79.1 ± 10.5	0.72

*Data are presented as mean ± standard deviation (SD)*.

*One-way ANOVA test used for assessment variables*.

** *P < 0.05*.

*FBG, fasting blood glucose; TG, triglyceride; HDL-C, high-density lipoprotein-cholesterol; SBP, systolic blood pressure; DBP, diastolic blood pressure; mg, milligram; dl, deciliter*.

The selected dietary intake of study participants across tertiles of CQI is shown in Supplementary Tables 2, 3. In habitual diet, we observed a significant association between tertiles in participants for total sugar, total fiber, GI (*P* < 0.001 for all), and carbohydrate (*P* = 0.001). Within breakfast meal, dietary intake of total sugar (*P* = 0.007), and GI (*P* < 0.001) were significantly different across tertiles of CQI. In lunch meals, dietary intakes of total fiber, total sugar, and GI were significantly different across tertiles of CQI among participants (*P* < 0.001 for all). Moreover, participants' carbohydrate intake (*P* = 0.02) and protein intake (*P* = 0.001) were significant across tertiles of CQI. Within the dinner meal, participants in the top of tertiles of CQI had a higher intake of energy and protein (*P* = 0.03; *P* = 0.02). In contrast, participants in the top of tertiles of CQI had a lower intake of fat and cholesterol (*P* = 0.01; *P* = 0.002). Moreover, dietary intakes of total fiber, total sugar, GI, and saturated fatty acids (SFA) (*P* < 0.001 for all).

The multivariate-adjusted odds ratio for MetS and its component across tertiles of habitual and meal-specific CQI is indicated in Tables 3, 4. In habitual diet and all three meals, we failed to find any significant association between tertiles of CQI and MetS either before or after adjustment for covariates. In habitual meal, before and after adjustment for the covariate, the odds ratio of low-HDL in third tertiles of CQI was significantly lower than the first tertiles [odds ratio (OR): 0.67; 95% CI: 0.47–0.96]. Moreover, within lunch meals, CQI is associated with low-HDL (OR: 0.66; 95% CI: 0.47–0.94) before and after adjustment for potential cofounding. In addition, no overall significant association was observed between CQI and other components of MetS either before or after adjustment for covariates in habitual diet and all the three meals. It should be noted that the trend of low-HDL across tertiles of CQI was marginally significant in habitual diet (OR: 0.67; 95% CI: 0.47–0.96; *P*-trend = 0.02) and in lunch meal (OR: 0.66; 95% CI: 0.47–0.94; *P*-trend = 0.01).

**Table 3:** Odds ratio (OR) and 95% CI for metabolic syndrome and components among the study participants according to tertiles (T) of CQI in habitual diet.

	**CQI**			
	**T1** **(3–7)**	**T2** **(7–11)**	**T3** **(11–15)**	***P*-trend**
Participant	276	277	276	
**Mets** ^†^				
Crude	1.00	0.74 (0.51–1.07)	1.05 (0.74–1.5)	0.76
Model 1	1.00	0.77 (0.52–1.13)	1.08 (0.74–1.54)	0.71
Model 2	1.00	0.83 (0.59–1.24)	1.14 (0.78–1.67)	0.52
Model 3	1.00	0.86 (0.57–1.29)	1.13 (0.76–1.69)	0.57
**Abdominal obesity**				
Crude	1.00	0.79 (0.56–1.1)	1.00 (0.72–1.4)	0.96
Model 1	1.00	0.81 (0.57–1.16)	1.07 (0.75–1.53)	0.7
Model 2	1.00	0.83 (0.57–1.19)	1.1 (0.76–1.58)	0.6
Model 3	1.00	0.79 (0.5–1.23)	1.15 (0.73–1.82)	0.55
**Elevated BP**				
Crude	1.00	0.8 (0.49–1.3)	1.05 (0.66–1.67)	0.8
Model 1	1.00	0.88 (0.53–1.46)	1.02 (0.63–1.66)	0.9
Model 2	1.00	0.83 (0.48–1.41)	0.98 (0.59–1.63)	0.98
Model 3	1.00	0.87 (0.51–1.5)	0.98 (0.59–1.65)	0.99
**Elevated FBG**				
Crude	1.00	0.96 (0.69–1.34)	1.35 (0.97–1.89)	0.07
Model 1	1.00	1.01 (0.72–1.43)	1.37 (0.97–1.93)	0.07
Model 2	1.00	1.02 (0.72–1.45)	1.4 (0.98–1.98)	0.05
Model 3	1.00	1.04 (0.73–1.47)	1.4 (0.98–1.99)	0.05
**Low HDL-C**				
Crude	1.00	0.95 (0.68–1.32)	0.63 (0.45–0.88)	0.008
Model 1	1.00	0.91 (0.65–1.28)	0.64 (0.46–0.91)	0.01
Model 2	1.00	1.02 (0.72–1.45)	0.67 (0.47–0.96)	0.02
Model 3	1.00	1.02 (0.72–1.45)	0.67 (0.47–0.96)	0.02
**Elevated TG**				
Crude	1.00	0.86 (0.61–1.22)	1.09 (0.77–1.54)	0.59
Model 1	1.00	0.89 (0.63–1.27)	1.04 (0.74–1.48)	0.79
Model 2	1.00	0.95 (0.66–1.36)	1.08 (0.75–1.54)	0.69
Model 3	1.00	0.97 (0.67–1.39)	1.08 (0.75–1.54)	0.7

*Binary logistic regression test used for assessment variables*.

*Crude: unadjusted model*.

*Model 1: adjusted for age, gender, and energy intake*.

*Model 2: In addition, adjusted for marital status, physical activity, education status, smoking, and underlying disease*.

*Model 3: further adjustment was made for BMI*.

† *Defined as the presence of ≥3 of the following components: abdominal obesity (waist circumference > 88 for women and > 102 for men); elevated blood pressure (BP ≥ 130/85 mmHg); elevated fasting blood glucose (FBG ≥ 100 mg/dl); low high-density lipoprotein-cholesterol (HDL-C < 50 for women and < 40 for men); elevated triglyceride (TG ≥ 150 mg/dl)*.

*^‡^Elevated blood pressure (systolic ≥ 130 and diastolic ≥ 85)*.

*Mets, metabolic syndrome; BP, blood pressure; FBG, fasting blood glucose; HDL-C, high-density lipoprotein cholesterol; TG, triglyceride*.

**Table 4:** OR and 95%CI for metabolic syndrome and components among the study participants according to tertiles (T) of CQI in meals.

	**CQI (breakfast)**				**CQI (lunch)**				**CQI (dinner)**			
	**T1**	**T2 7–11)**	**T3**	P–trend**	**T1**	**T2**	**T3**	P–trend**	**T1**	**T2**	**T3**	P–trend**
	**(3–7)**	**(7–11)**	**(11–15)**		**(3–7)**	**(7–11)**	**(11–15)**		**(3–7)**	**(7–11)**	**(11–15)**	
Participant	273	274	273		275	276	275		281	282	282	
**Mets** ^†^												
Crude	1.00	0.77 (0.54–1.1)	0.8 (0.56–1.14)	0.21	1.00	26.1 (0.89–1.8)	0.92 (0.64–1.33)	0.69	1.00	0.89 (0.62–1.27)	0.96 (0.68–1.37)	0.85
Model 1	1.00	0.8 (0.55–1.15)	0.8 (0.56–1.16)	0.25	1.00	1.25 (0.86–1.8)	0.92 (0.63–1.34)	0.68	1.00	0.83 (0.57–1.21)	0.86 (0.59–1.25)	0.44
Model 2	1.00	0.86 (0.58–1.26)	0.79 (0.54–1.16)	0.23	1.00	1.32 (0.9–1.94)	0.9 (0.61–1.34)	0.62	1.00	0.85 (0.58–1.25)	0.9 (0.61–1.31)	0.53
Model 3	1.00	0.87 (0.58–1.29)	0.81 (0.55–1.21)	0.32	1.00	1.23 (0.83–1.83)	0.86 (0.58–1.29)	0.48	1.00	0.87 (0.58–1.29)	0.96 (0.65–1.43)	0.8
**Abdominal obesity**												
Crude	1.00	0.84 (0.6–1.17)	0.87 (0.63–1.22)	0.44	1.00	1.64 (1.17–2.3)	1.31 (0.94–1.84)	0.1	1.00	0.95 (0.68–1.33)	0.88 (0.63–1.23)	0.46
Model 1	1.00	0.88 (0.61–1.25)	0.91 (0.63–1.3)	0.61	1.00	1.63 (1.14–2.34)	1.34 (0.93–1.93)	0.1	1.00	0.87 (0.61–1.25)	0.8 (0.55–1.14)	0.22
Model 2	1.00	0.89 (0.61–1.28)	0.9 (0.62–1.29)	0.57	1.00	1.65 (1.14–2.39)	1.31 (0.91–1.89)	0.1	1.00	0.89 (0.61–1.28)	0.8 (0.56–1.16)	0.25
Model 3	1.00	0.85 (0.54–1.33)	0.94 (0.61–1.47)	0.83	1.00	1.56 (1–2.44)	1.3 (0.83–2.04)	0.2	1.00	0.89 (0.56–1.39)	0.81 (0.51–1.26)	0.34
**Elevated BP**												
Crude	1.00	0.7 (0.43–1.15)	1.12 (0.71–1.76)	0.58	1.00	1.19 (0.74–1.9)	1.08 (0.67–1.75)	0.74	1.00	1.04 (0.65–1.65)	0.89 (0.55–1.44)	0.65
Model 1	1.00	0.71 (0.43–1.19)	1.15 (0.72–1.84)	0.54	1.00	1.22 (0.74–1.99)	1.1 (0.66–1.81)	0.7	1.00	0.96 (0.59–1.56)	0.73 (0.44–1.21)	0.22
Model 2	1.00	0.8 (0.47–1.37)	1.1 (0.67–1.8)	0.7	1.00	1.22 (0.73–2.04)	1.12 (0.67–1.88)	0.66	1.00	0.98 (0.59–1.63)	0.69 (0.41–1.17)	0.18
Model 3	1.00	0.85 (0.49–1.45)	1.18 (0.71–1.95)	0.51	1.00	1.17 (0.69–1.98)	1.13 (0.67–1.91)	0.64	1.00	0.99 (0.59–1.66)	0.73 (0.43–1.24)	0.25
**Elevated FBG**												
Crude	1.00	0.83 (0.59–1.15)	0.83 (0.6–1.16)	0.29	1.00	1.00 (0.72–1.4)	0.93 (0.67–1.3)	0.7	1.00	1.04 (0.74–1.45)	1.23 (0.88–1.72)	0.2
Model 1	1.00	0.84 (0.6–1.18)	0.85 (0.61–1.2)	0.37	1.00	0.99 (0.7–1.39)	0.93 (0.66–1.3)	0.67	1.00	0.97 (0.68–1.36)	1.14 (0.81–1.6)	0.44
Model 2	1.00	0.88 (0.62–1.24)	0.88 (0.62–1.24)	0.47	1.00	0.97 (0.68–1.36)	0.92 (0.65–1.29)	0.66	1.00	0.95 (0.67–1.35)	1.13 (0.8–1.6)	0.42
Model 3	1.00	0.88 (0.62–1.24)	0.88 (0.63–1.25)	0.5	1.00	0.94 (0.66–1.33)	0.91 (0.64–1.29)	0.62	1.00	0.96 (0.68–1.36)	1.16 (0.82–1.64)	0.37
**Low HDL-C**												
Crude	1.00	0.81 (0.58–1.14)	0.97 (0.7–1.36)	0.9	1.00	0.95 (0.68–1.32)	0.69 (0.5–0.97)	0.03	1.00	0.92 (0.66–1.29)	1.1 (0.79–1.53)	0.55
Model 1	1.00	0.81 (0.58–1.14)	0.97 (0.69–1.35)	0.86	1.00	0.91 (0.65–1.28)	0.68 (0.48–0.95)	0.02	1.00	0.9 (0.64–1.27)	1.13 (0.81–1.58)	0.46
Model 2	1.00	0.8 (0.56–1.13)	0.97 (0.69–1.37)	0.86	1.00	0.98 (0.69–1.38)	0.66 (0.47–0.94)	0.02	1.00	0.97 (0.68–1.37)	1.21 (0.85–1.71)	0.38
Model 3	1.00	0.8 (0.56–1.13)	0.97 (0.69–1.37)	0.86	1.00	0.97 (0.69–1.38)	0.66 (0.47–0.94)	0.01	1.00	0.97 (0.69–1.38)	1.21 (0.86–1.72)	0.37
**Elevated TG**												
Crude	1.00	0.87 (0.62–1.23)	0.78 (0.55–1.1)	0.16	1.00	0.93 (0.66–1.31)	0.79 (0.56–1.11)	0.18	1.00	0.94 (0.67–1.33)	1.1 (0.78–1.55)	0.55
Model 1	1.00	0.88 (0.62–1.24)	0.79 (0.56–1.12)	0.19	1.00	0.95 (0.67–1.34)	0.8 (0.56–1.13)	0.21	1.00	0.93 (0.65–1.32)	1.03 (0.72–1.45)	0.86
Model 2	1.00	0.94 (0.66–1.34)	0.81 (0.57–1.16)	0.26	1.00	0.96 (0.68–1.37)	0.78 (0.55–1.12)	0.18	1.00	0.92 (0.64–1.31)	1.04 (0.73–1.48)	0.83
Model 3	1.00	0.94 (0.66–1.34)	0.82 (0.58–1.18)	0.29	1.00	0.93 (0.65–1.33)	0.77 (0.54–1.11)	0.16	1.00	0.93 (0.65–1.33)	1.07 (0.75–1.52)	0.73

*Binary logistic regression test used for assessment variables*.

*Crude: Unadjusted model*.

*Model 1: Adjusted for age, gender, and energy intake*.

*Model 2: In addition, adjusted for marital status, physical activity, education status, smoking, and underlying disease*.

*Model 3: further adjustment was made for BMI*.

** *P < 0.05*.

† *Defined as the presence of ≥3 of the following components: abdominal obesity (waist circumference > 88 for women and > 102 for men); elevated blood pressure (BP ≥ 130/85 mmHg); elevated fasting blood glucose (FBG ≥ 100 mg/dl); low high-density lipoprotein-cholesterol (HDL-C < 50 for women and < 40 for men); elevated triglyceride (TG ≥ 150 mg/dl)*.

*^‡^Elevated blood pressure (systolic ≥ 130 and diastolic ≥85)*.

*Mets, metabolic syndrome; BP, blood pressure; FBG, fasting blood glucose; HDL-C, high-density lipoprotein cholesterol; TG, triglyceride*.

## Discussion

This study examined the relationship between habitual- and meal-specific CQI and odds of MetS and its components in a sample of Iranian adults. Our findings showed a nonsignificant association between CQI with MetS before and after adjustment for potential confounders in all three meals and habitual diet. But we found a reverse association between CQI and the odds of developing low-HDL in habitual diet and within lunch meal even after adjustment for covariates. No overall significant association was observed between CQI and other components of MetS in habitual diet and all three meals.

Carbohydrates are a heterogeneous class of nutrients and the consumption of refined carbohydrates for enhancing the quality of received carbohydrates from a public health perspective has been declared. In this way, the available studies on adults demonstrate that total carbohydrate intake or dietary carbohydrate proportion is not associated with the risk of obesity (38, 39). According to a report from a population, carbohydrate quality is a more important factor compared to the fat quality to determine diet quality (40) as far as, it has been proposed that a decline in fat intake was compensated with an enhancement intake of refined starches and sugars (41). There is convincing evidence that carbohydrate quality has important effects on the advancement and treatment of CVD, MetS, T2D, and obesity (42). Aspects of carbohydrate quality that may be substantial in these components include dietary fiber, whole-grain, GI, and GL, particularly the intake of sugar-sweetened drinks. However, their properties are often highly interrelated, and it may be hard to implicate one over another in any specific condition (42). Therefore, in this context, this seems to be more important in Iranian populations because of the higher intake of carbohydrates (43).

In line with our findings in habitual diets, a cross-sectional study conducted on Korean adults observed no significant associations between CQI and T2DM or MetS. However, the quality of carbohydrates consumed is associated with the risk of obesity and elevated blood pressure (44). Also, in another study, no association was found between GI, GL, and MetS (45). Contrary to our findings, a cross-sectional study from Ghana demonstrates that the diet with high CQI levels is inversely related to general and abdominal obesity (25). Another study identified that consuming refined foods with high carbohydrate content was a direct association with a higher risk of abdominal obesity in Ghanaian University students (46). A cohort study from Spain in university graduates reported an inverted association between dietary CQI and general obesity (24). The result of a systematic review and meta-analysis study focusing on the association between carbohydrate quality and NCDs incidence and metabolic biomarkers demonstrated that daily consumption of dietary fiber was associated with a reduced risk of health-related consequences. These findings are supported by cohort studies, which report an alleviated risk of coronary heart disease, mortality, and occurrence of diabetes (47).

High carbohydrate diets, which are common in developing nations, especially Asian countries, contain a high content of refined sources (such as white rice and white bread), low in fiber. These diets usually reflect poor food quality and mainly have high GI content, which can lead to negative metabolic outcomes (48–50). According to the National Food Consumption Survey, most of the calorie intake in Iranian people, which is about more than 60%, is obtained from carbohydrates. In other words, the amount of carbohydrates in Iranians diet is 450 g per day (rural areas: 413 g/day and urban areas: 518 g/day) (51). In addition, considering the high prevalence of low HDL-c in both sexes of the Iranian people (43%; 95% CI: 33–53) (52), the results of our study can be effective as a strategy in controlling the optimal level of low-HDL. The present observation of a reverse association between CQI and low HDL-c is in consent with previous studies. The study that indicated the relation between carbohydrate quality and the prevalence of MetS showed no association between GI, GL, and MetS. However, these were positively associated with low HDL-c levels in adults and older adults (45). Another study found a positive association between GI, GL, and low HDL-c in women (22).

Meanwhile, Data from NHANES III (1988–1994), the Cooper Center Longitudinal Study, reported a positive link between GL and low HDL-c in males and females (53, 54). The exact mechanism of this association is not known yet. When dietary GI and GL content is high, digestion and absorption of food are done at high speed, resulting in high blood sugar and consequent hyperinsulinemia release. These adjustments are made by regulatory hormones (cortisol, glucagon, and growth hormone), reducing reactive hypoglycemia and enhancing the secretion of free fatty acid (55). The metabolic response by increasing satiety and decreasing fat storage can lead to obesity. Excess body fat and the increased release of free fatty acids could contribute to the development of dyslipidemia, containing low HDL-c levels. In addition, diets with high GI/GL could cause insulin resistance, oxidative stress, and chronic inflammation aggravating dyslipidemia (56, 57).

Insulin resistance as a predictor factor of MetS, one of the main complications of chronic inflammation and stress oxidative can be considered. Particular attention has been paid to the quantitative characteristics of dose-response relationships and the underlying mechanisms that can investigate the nature of biphasic hormonal responses after exposure to redox-active agents, such as free radical oxygen species and identify their effect on inflammatory and anti-inflammatory pathways (58). Understanding that hormesis may prolong life and reduce the incidence of chronic diseases involves the optimal challenge of cells and entire organisms by any of the broader stressors, including pharmacological, physical, dietary, exercise, and ischemic (59). Antiaging and neuroprotective effects of hormetic have been reported using experimental protocols in a wide range of *in vitro* and *in vivo* models (60, 61). Among foods, several bioactive compounds, including polyphenols, have been considered as health promoters. Polyphenols include four main classes of flavonoids, phenolic acids, acetylbones, and lignans, each of which has different effects (62). Polyphenols with anti-inflammatory and antioxidant effects are a great choice to improve the diet quality (63). Polyphenols regulate cellular and enzymatic activities involved in inflammatory pathways by preventing the overproduction of reactive oxygen species and inhibiting free radicals (64). In addition to reducing apoptosis, promoting pancreatic β-cell proliferation, are involved in glucose homeostasis (65).

However, previous studies did not examine the relationship between meals. Based on our knowledge, there has been no observational study examining the relationship between carbohydrate quality in meals and metabolic disorders. Dietary approaches derived from meal timing are hopeful for modulating circadian rhythms and clock-controlled metabolic functions in humans. Besides, studies have proposed that specific times are more suitable for consuming carbohydrate-rich or fat-rich food to maintain metabolic health. A crossover trial investigated that consumption of high-carb meals in the evening undesirable influences blood glucose level and glycemic control in individuals with impaired glucose metabolism (66). In agreement with this finding, other studies in humans suggested that a carbohydrate-rich diet at the beginning of the day could be safe vs. the development of diabetes and MetS (67, 68). In addition, the inclusion of lunch as the main meal, compared to dinner, seems to have beneficial effects on reducing MetS risk factors (69).

Western main meals usually end with a sweet dessert. In addition, drinking beverages during the main meal was considered part of modern lifestyle; also, the food eaten, especially at breakfast, is mainly different from the foods eaten at other meals (70). On the other hand, skipping breakfast is very common in modern societies. In a study of eight young men, participants ate three main meals (breakfast, lunch, and dinner), while in the other condition, the same amount of energy was consumed at lunch and dinner times only. They found that skipping breakfast enhanced the blood glucose concentration during the afternoon and sleep and increased 24-h average blood glucose concentration (71). Another study assessed the glucose metabolism of healthy adults in two conditions of breakfast and dinner skipping. They showed breakfast skipping conduced higher glucose concentrations and insulin resistance after lunch (72). Similarly, our study highlights the importance of meals, especially lunch meals. In this way, if the lunch meal has a good CQI (for example, it is prepared from whole grains and fruits and vegetables with low GI and GL), it can play a role in controlling and managing the normal level of HDL.

The content of dietary fiber and vitamin minerals in whole grains is higher than in refined carbohydrates. The protective effects of these nutrients vs. the risk of chronic diseases are well-known (73, 74). Due to their physical structure and dietary fiber content, whole grains are categorized as low GI foods (34). The use of whole grains is a useful way to increase fiber in the diet and reduce noncommunicable diseases (NCDs). In addition, fruits and vegetables are important factors in fiber intake in the diet. According to the above, our calculated CQI has low quality. Still, due to the consumption of medium to the high fiber content in this study, it can be concluded that the intake of fruits and vegetables and solid carbohydrates in the diet of individuals are higher. Based on the considerable role of carbohydrates in Iranian diets and their low-quality diets, focusing on improving carbohydrate quality would be a beneficial strategy to make better food choices among Iranians (43).

In prospective studies, liquid carbohydrates intake was associated with weight gain, while there was a reverse association between consumption of solid carbohydrates and high weight gain (75–78). Examination of the systematic evidence presented for the effect of long-term intervention with low GI and GL on fasting insulin level and proinflammatory markers showed that it could effectively prevent obesity-related disease (79).

Dietary fiber and whole grains are more related to health outcomes than the GI or GL content of foods. Although the GI provides a measure of the glycemic potential of the carbohydrate content of foods, some low GI foods might have other attributes that are not health promoting. Foods with added fructose or sucrose and mixed foods high in SFA and carbohydrate (i.e., confectionery products) may have a low GI (33). Findings from a dose-response meta-analysis showed that diets identified by low dietary fiber contribute to NCDs, and therefore, quantitative recommendations for dietary fiber intake will be beneficial. While consumption in the range of 25–29 g/day is sufficient, dose-responses data showed that amounts >30 g/day have more advantages (47).

Given the effect of low GI in the development of obesity, there is evidence that low GI diets enhance satiety by reducing voluntary food intake, thus reducing total energy intake. It can be effective for body-weight maintenance. It may prevent obesity (80–82). In contrast, the intake of a high GI diet motivates increases in hunger and leads to increased food intake, thus affecting energy balance and body composition (83). Fiber-containing foods should be chewed before passing through the stomach and into the small bowel, affecting satiety, glucose and insulin responses, and lipid absorption. Whole foods that require chewing and retain much of their structure in the gut are more likely to cause a feeling of satiety, leading to weight loss and balance of carbohydrate and lipid metabolism. In the large bowel, fiber is almost completely broken down by the resident microflora under a set of anaerobic reactions known as fermentation. The gut microbiota plays a substantial role in human health (84).

This study has important strengths. To the authors' knowledge, this study was the first to investigate CQI in meals and its association with MetS and its components. We had a sufficient sample size in this study that was done within 25 health houses in the Tehran Metropolis. Despite these strengths, the study has some limitations. First, this study had a cross-sectional design, and the findings do not establish causality between CQI and MetS; therefore, the results should be interpreted with caution. Second, using the questionnaire retrospectively may reduce information recall. There was also under-reporting and over-reporting of food items received. Third, even though the data were controlled for some potential confounders, the effects of eating behavior, menopausal status, and residual confounding cannot be discounted.

## Conclusion

In conclusion, in this study, CQI was not associated with MetS. However, CQI may contribute to the nutritional therapy of improving low-HDL. Although, findings should be treated with caution, considering several conflicting results between studies. Further investigations into the mechanisms underlying the role of carbohydrate quality in the development of metabolic disorders are warranted.

## Data Availability Statement

The raw data supporting the conclusions of this article will be made available by the authors, without undue reservation.

## Ethics Statement

The studies involving human participants were reviewed and approved by Tehran University of Medical Sciences (Ethic Number: IR.TUMS.MEDICINE.REC.1399.797). The patients/participants provided their written informed consent to participate in this study.

## Author Contributions

SS-B and KD contributed to the conception/design of the research and critically revised the manuscript. MM and ZA contributed to the acquisition of data. MM, FH, and EB participated in the analysis and interpretation of the data. MM, AL, and HI drafted the manuscript. SS-B agreed to be fully accountable for ensuring the integrity and accuracy of the work. All authors contributed to manuscript revision, read, and approved the submitted version of the manuscript.

## Conflict of Interest

The authors declare that the research was conducted in the absence of any commercial or financial relationships that could be construed as a potential conflict of interest.

## Publisher's Note

All claims expressed in this article are solely those of the authors and do not necessarily represent those of their affiliated organizations, or those of the publisher, the editors and the reviewers. Any product that may be evaluated in this article, or claim that may be made by its manufacturer, is not guaranteed or endorsed by the publisher.
