# Supplementary material for: Habitual- and Meal-Specific Carbohydrate Quality Index and Their Relation to Metabolic Syndrome in a Sample of Iranian Adults
Source: Front Nutr. 2022 Apr 1;9:763345. doi: 10.3389/fnut.2022.763345 (PMC9011184; doi:10.3389/fnut.2022.763345)
Supplement: Supplementary file 1 [file Data_Sheet_1.docx]

**Supplementary Figure 1: Sample composition flow**

**Supplementary Table 1: General characteristics of study participants**

|  |  | | |
| --- | --- | --- | --- |
| Characteristics | **Without MetS** | **With MetS** | **P-value^**^** |
| Age | 40.2±10.28 | 46.1±10 | <0.001* |
| BMI (kg/m^2^) | 26.2±4.07 | 29.2±4.71 | <0.001* |
| Physical activity |  |  |  |
| Low | 65.1 | 34.9 | 0.1 |
| Moderate | 69.7 | 30.3 | 0.1 |
| High | 75 | 25 | 0.1 |
| WC | 85.810.6 | 95.710.8 | <0.001* |
| SBP (mmHg) | 114.1±13 | 126±16.5 | <0.001* |
| DBP (mmHg) | 76.5±8.04 | 83.3±11.3 | <0.001* |
| FBG (mg/dl) | 100.2±22.2 | 123.7±50.1 | <0.001* |
| TC (mg/dl) | 192.7±43.2 | 203.7±47.6 | 0.001* |
| TG (mg/dl) | 117.3±55.5 | 203±85.4 | <0.001* |
| HDL-C (mg/dl) | 52.2±10.1 | 44.9±8.41 | <0.001* |
| Total energy (kcal) | 1628.6±403.3 | 1615.6±363.3 | 0.6 |
| CQI | 8.91±2.88 | 9.15±2.83 | 0.2 |

Values are means (standard deviations) or percentages

† Student t-test used for continuous variables; Chi-square test used for categorical variables

** *P* < 0.05

Abbreviation: *BMI* body mass index; *WC* waist circumference; *SBP* systolic blood pressure; *DBP* diastolic blood pressure; *FBG* fasting blood glucose; *TC* total cholesterol; *TG* triglyceride; *HDL-C* high-density lipoprotein-cholesterol; *CQI* carbohydrate quality index

*kg/m^2^* kilogram/meter^2^*; mg* milligram; *dl* deciliter; *kcal* kilocalorie

**Supplementary Table 2: Dietary intake of study participants according to tertiles (T) of Carbohydrate Quality Index (CQI) in habitual diet**

|  | CQI | | |  |
| --- | --- | --- | --- | --- |
|  | **T1**  **(3-7)** | **T2**  **(7-11)** | **T3**  **(11-15)** | **P-value**** |
| Participant | **273** | **274** | **273** |  |
| Total energy (kcal) | 1599.9 ± 352.8 | 1662.2 ± 307.5 | 1620.5 ± 378.2 | 0.09 |
| Carbohydrate (g/d) | 230.1 ± 52 | 240.5 ± 57.11 | 251 ± 85.72 | 0.001 |
| Protein (g/d) | 55.75 ± 15.15 | 59.07 ± 25.02 | 58.8 ± 20.68 | 0.11 |
| Fat (g/d) | 55.57 ± 27.69 | 55.04 ± 17.22 | 56.05 ± 18.58 | 0.86 |
| SFA (g/d) | 15.08 ± 6.42 | 15.26 ± 4.44 | 15.64 ± 7.31 | 0.55 |
| MUFA (g/d) | 15.67 ± 5.73 | 17.73 ± 11.91 | 19.29 ± 31.35 | 0.09 |
| PUFA (g/d) | 16 ± 4.85 | 17.08 ± 6.69 | 17.2 ± 7.88 | 0.06 |
| Cholesterol (mg/d) | 213.9 ± 109.3 | 202.7 ± 96.9 | 204.1 ± 110.02 | 0.39 |
| Total fiber (g/d) | 15.59 ± 3.54 | 19.93 ± 11.61 | 35.07 ± 58.83 | <0.001 |
| Total sugar (g/d) | 38.61 ± 13.21 | 42.72 ± 21.5 | 57.72 ± 67.13 | <0.001 |
| Glycemic Index | 62.43 ± 2.55 | 60.59 ± 3.62 | 59 ± 3.73 | <0.001 |

Data are presented as mean ± standard deviation (SD).

* One-way ANOVA test used for assessment variables.

**P-value < 0.05

Abbreviations: *SFAs* saturated fatty acids, *MUFAs* mono-unsaturated fatty acids, *PUFAs* poly-unsaturated fatty acids *Kcal* kilocalorie, *g* gram, *d* day

**Supplementary Table 3: Dietary intake of study participants according to tertiles (T) of Carbohydrate Quality Index (CQI) in meals**

|  | CQI (breakfast) | | | | CQI (lunch) | | | | CQI (dinner) | | | |
| --- | --- | --- | --- | --- | --- | --- | --- | --- | --- | --- | --- | --- |
|  | **T1**  **(3-7)** | **T2**  **(7-11)** | **T3**  **(11-15)** | **P^**^** | **T1**  **(3-7)** | **T2**  **(7-11)** | **T3**  **(11-15)** | **P^**^** | **T1**  **(3-7)** | **T2**  **(7-11)** | **T3**  **(11-15)** | **P^**^** |
| Participant | **273** | **274** | **273** |  | **275** | **276** | **275** |  | **281** | **282** | **282** |  |
| Total energy (kcal) | 415.9 ± 139.4 | 432.8 ± 161.9 | 408 ± 158.5 | 0.14 | 537.3 ± 198.2 | 516.8 ± 154.4 | 552.9 ± 178.7 | 0.05 | 492.8 ± 182.3 | 533.9 ± 202.5 | 502.4 ± 193.9 | 0.03 |
| Carbohydrate (g/d) | 71.61 ± 26.78 | 72.74 ± 28..79 | 68.96 ± 33.03 | 0.29 | 72.22 ± 46.31 | 65.02 ± 23.92 | 64.99 ± 32.03 | 0.02 | 70.57 ± 29.94 | 72.58 ± 27.18 | 75.16 ± 44.6 | 0.29 |
| Protein (g/d) | 12.37 ± 5.23 | 14.79 ± 22.44 | 12.87 ± 14.12 | 0.15 | 23.56 ± 9.28 | 21.44 ± 8.36 | 21.15 ± 8.04 | 0.001 | 17.75 ± 7.55 | 19.55 ± 7.94 | 19.11 ± 8.72 | 0.02 |
| Fat (g/d) | 13.75 ± 22.4 | 13.25 ± 8.16 | 12.83 ± 8.56 | 0.75 | 21.01 ± 9.62 | 20.01 ± 9.61 | 19.78 ± 8.71 | 0.25 | 17.25 ± 8.32 | 18.6 ± 9.65 | 16.52 ± 8.08 | 0.01 |
| SFA (g/d) | 5.83 ± 2.67 | 5.44 ± 2.55 | 5.92 ± 6.4 | 0.36 | 5.2 ± 3.23 | 4.85 ± 2.63 | 4.87 ± 3.96 | 0.38 | 4.5 ± 2.22 | 5.19 ± 2.78 | 4.21 ± 2.61 | <0.001 |
| MUFA (g/d) | 4.07 ± 2.27 | 3.74 ± 2.4 | 3.89 ± 6.09 | 0.61 | 7.01 ± 5.25 | 7.22 ± 10.63 | 7.24 ± 4.24 | 0.91 | 7.93 ± 30.01 | 6.31 ± 3.39 | 5.23 ± 3.01 | 0.18 |
| PUFA (g/d) | 2.43 ± 1.72 | 2.23 ± 1.87 | 2.71 ± 6.08 | 0.33 | 7.86 ± 4.1 | 7.72 ± 2.94 | 7.35 ± 2.92 | 0.18 | 6.5 ± 2.88 | 7.01 ± 5.32 | 6.38 ± 3.36 | 0.13 |
| Cholesterol (mg/d) | 57.39 ± 50.74 | 65.16 ± 60.09 | 63.49 ± 60.12 | 0.23 | 69.77 ± 60.86 | 72.23 ± 61.41 | 68.8 ± 63.45 | 0.79 | 83.39 ± 70.88 | 76.81 ± 61.74 | 64.44 ± 62.73 | 0.002 |
| Total fiber (g/d) | 7.23 ± 42.69 | 8.57 ± 32.2 | 10.13 ± 25.25 | 0.59 | 4.93 ± 2.25 | 6.85 ± 3.31 | 9.88 ± 5.41 | <0.001 | 4.46 ± 1.61 | 7.02 ± 2.1 | 11.35 ± 8.49 | <0.001 |
| Total sugar (g/d) | 9.73 ± 8.21 | 8.51 ± 8.03 | 7.6 ± 8.003 | 0.007 | 22.91 ± 39.28 | 13.56 ± 35.42 | 3.07 ± 18.43 | <0.001 | 18.46 ± 36.58 | 15.17 ± 35.58 | 3.74 ± 15.02 | <0.001 |
| Glycemic Index | 65.04 ± 4.61 | 62.75 ± 7.14 | 58.73 ± 9.28 | <0.001 | 62.77 ± 2.71 | 60.89 ± 3.76 | 59.18 ± 4.4 | <0.001 | 62.3 ± 3.53 | 58.99 ± 5.52 | 55.41 ± 6.7 | <0.001 |

Data are presented as mean ± standard deviation (SD).

* One-way ANOVA test used for assessment variables.

**P < 0.05 Abbreviations: *SFAs* saturated fatty acids, *MUFAs* mono-unsaturated fatty acids, *PUFAs* poly-unsaturated fatty acids *Kcal* kilocalorie, *g* gram, *d* day
